# Beyond Self-Identification: Rethinking Alcohol Risk Detection and Pathways in UK Adults

**DOI:** 10.1192/bjo.2026.11084

**Published:** 2026-06-30

**Authors:** Tessa Lapelyte, David McLaughlan, Anthony Eadon, Aaron Brown, Faith Matcham

**Affiliations:** 1 University of Sussex, Brighton, United Kingdom; 2 Clean Slate Clinic, London, United Kingdom

## Abstract

**Aims:**

Alcohol harm in the UK imposes a substantial clinical and societal burden, with 339,916 alcohol-specific hospital admissions recorded in 2023/24. Current pathways for identifying and supporting people at risk rely on self-identification of alcohol use.The aim of this work is to examine the extent to which self-identification as a “heavy drinker” aligns with clinically defined alcohol-risk levels and to identify the structural, stigma-related, and systemic barriers that prevent higher-risk drinkers from accessing appropriate support.

**Methods:**

This analysis draws on nationally representative polling of 2,037 UK adults (mean age 48.77, SD=17.74, 52.33% female) applying the Alcohol Use Disorders Identification Test-Consumption (AUDIT-C) scoring to examine clinical risk, self-perception of drinking identity, barriers to accessing support, and systemic implications for service design.

**Results:**

Among adults meeting AUDIT-C criteria for increasing or higher risk (25.8%), 90% did not self-identify as a heavy drinker, with most describing themselves as “moderate” or “occasional” drinkers. This disconnect challenges the continuing reliance on self-referral and identity-based messaging within NHS and workplace pathways. Help-seeking was shaped primarily by systemic barriers: long NHS wait times (24.5%), stigma (24.1%), and the cost of private care (19.4%), while “not knowing where to go for help” ranked only sixth (16.9%). These findings contradict policy assumptions that awareness deficits are the primary obstacle. Exposure to alcohol harm extended far beyond the drinker: 49.3% of UK adults knew someone they considered a heavy drinker, indicating significant family, peer, and workplace impact and highlighting a missed early-intervention opportunity within social networks.

**Conclusion:**

Self-identification is not a reliable gateway to care; stigma-laden service framing actively excludes the majority at clinical risk; and capacity constraints limit timely support even when motivation exists. These findings highlight the need for a shift toward routine use of the AUDIT-C in primary care, workplace health initiatives, and NHS Health Checks so that alcohol-risk detection no longer depends on individuals self-identifying as “heavy drinkers”. Services should adopt identity-neutral language, such as referring to “supported reduction” or “health optimisation”, to reduce the stigma that prevents many higher-risk drinkers from seeking help. Supporting families, friends, and colleagues to play a constructive role in early recognition and intervention when they observe escalating risk, is critical. Commissioning should prioritise rapid-access and digitally enabled models of support, to reduce long wait times and make care more accessible and discreet for people who face barriers related to work, geography, or stigma.

